# Occlusal Features and Caries Experience of Hong Kong Chinese Preschool Children: A Cross-Sectional Study

**DOI:** 10.3390/ijerph14060621

**Published:** 2017-06-09

**Authors:** Shinan Zhang, Edward Chin Man Lo, Chun Hung Chu

**Affiliations:** 1 School of Stomatology, Kunming Medical University, Yunnan 650031 China; snzhang2000@163.com; 2 Faculty of Dentistry, The University of Hong Kong, Hong Kong, China; edward-lo@hku.hk

**Keywords:** occlusal features, malocclusion, caries, children, primary teeth

## Abstract

*Objective:* To study occlusal features and their relation to caries experience in Hong Kong Chinese preschool children. *Methods:* Seven kindergarten classes in Hong Kong were selected using a stratified random sampling method, and the 4- and 5-year-old Chinese kindergarten children were invited to join the survey. Two calibrated dentists performed the clinical examinations in the kindergartens. Occlusion features, including incisal overjet; canine and molar relationship; median diastema; and primate space as well as malocclusion features, including crossbite; anterior open bite; and scissor bite, were recorded. Caries experience was recorded with the dmft index. *Results:* A total of 538 children were invited to participate in the study, and finally 495 children were examined (92%). Approximately half (52%) had a normal incisor relationship. Most children had a class I canine relationship (left-79%; right-75%). Approximately two-thirds had a flush terminal plane (left-64%; right-65%). Most children (76%) had a maxillary primate space, and 47% had a mandibular primate space. The prevalence of median diastema, anterior crossbite, and unilateral posterior crossbite was 30%, 12% and 1%, respectively. No bilateral posterior crossbite or scissor bite was found. Approximately half (49%) had caries experience. The mean dmft score was 2.1. Gender and age were not associated with either the studied occlusal features or the mean dmft score (*p* > 0.05). *Conclusion:* Approximately half of the Hong Kong preschool children studied had a normal occlusion, and two-thirds of them had a flush terminal plane. About three-quarters of them had a maxillary primate space, and half of them had a mandibular primate space. Their occlusal traits were not associated with caries experience and prevalence.

## 1. Introduction

Many studies on primary dentition in children have focused on reporting dental anomalies, such as supernumeraries [1], mesiodens [2], hypodontia [3], taurodontism [4], dens in dente [5], talon cusp [6], dysplasia [7], fusion [8], and double teeth [9]. Malocclusion in the primary teeth is not uncommon, and it usually stems from environmental factors, such as digit sucking and trauma. However, few studies have reported on malocclusion or occlusal characteristics in the primary dentition of children. Few studies have also investigated the changes in the occlusal features of the primary teeth during growth and development. The establishment and maintenance of normal and functional occlusion constitutes one of the most important objectives of paediatric dentistry [10].

Characteristics of malocclusion in primary teeth might be found in the succeeding permanent dentition [11]. The malocclusion reported can vary significantly with the population studied [10]. Although the assessment method and notation might differ between studies, wide variations among children may exist due to individual oral habits and tooth wear. A study on occlusal status and the prevalence of occlusal malocclusion traits among schoolchildren found a wide range of orthodontically relevant occlusal traits [12]. The authors concluded that orthodontic screening at 9 years of age or earlier should be conducted to monitor the growth and development of the dentition. Some studies reported mixed or permanent dentition [13,14,15]. The recognition of normal occlusion patterns and the identification of morphological changes in mixed dentition is essential for treatment planning in paediatric dentistry and for the planning of interceptive orthodontic treatment [10]. 

Occlusal features in primary dentition can be studied in the arrangement of maxillary and mandibular teeth and in the occlusal relationship of the anterior and posterior segments. A literature search on PubMed using the keywords of ((malocclusion OR occlusion OR occlusal features) and (primary teeth or primary dentition) and (Hong Kong)) from 2000 to 2017 was conducted. No published study focused on dental caries and its relation to occlusion in Hong Kong children. The association between occlusal traits and caries experience in the primary dentition of Hong Kong preschool children was unclear. Therefore, the aim of this study was to examine the occlusal features and their relation to caries experience in the primary dentition of southern Hong Kong children who were 4 to 5 years old at the time of study. 

## 2. Methods

Approval was sought from an independent review board at the University of Hong Kong (IRB 09-302). As most preschool children attend kindergarten, recruitment of the participants was performed through kindergarten classes. The list of all registered kindergarten classes was obtained from the Education Bureau of the Hong Kong Government, and a stratified sampling method was adopted. Hong Kong has three main geographical regions: the New Territories, Kowloon, and Hong Kong Island. Kindergarten classes were sampled in proportion to the population of the respective region [16]. Seven kindergartens were ultimately invited, and all agreed to take part in the study. All 4- and 5-year-old children of these seven kindergartens were invited to join the survey. The parents of the children received an invitation letter with the survey details. In addition, written consent was obtained before the survey. Children with serious systemic health problems, such as congenital heart disease, were excluded. 

This survey dovetailed with an epidemiological study of the caries experience [17]. The sample size was determined with reference to a previously published study [18]. In that study, the mean dmft score of Hong Kong preschool children was 1.6 ± 2.9. With a maximum expected 95% confidence interval of 0.15 on both sides, the minimum required sample size was 495. The response rate was expected to be 80%, and hence, at least 619 preschool children had to be invited. The details of caries status and its associated factors are reported elsewhere [17]. This study reports the occlusal features and their association with caries experience. Demographic data (gender and age) were used to correlate with the studied occlusion features.

Calibration was carried out by two trained dentists in the kindergartens before the main study. Twenty 5-year-old children with a full range of expected conditions were preselected. The examiner examined the 20 preschool children twice for a time interval of around 30 min. Their kappa static was above 0.9. Disposable dental mirrors attached to intraoral light-emitting diode (LED) lights were used, and ball-ended Community Periodontal Index (CPI) probes were used to remove food debris. Caries experience was recorded using the criteria recommended by the World Health Organization (WHO) [19]. Caries was diagnosed when a lesion had an unmistakable cavity, undermined enamel, or a detectably softened floor or wall. The dmft index was used to record the caries experience of the primary dentition: “d” stood for a decayed tooth, “m” denoted a missing tooth due to decay, and “f” represented a filled tooth. No dental radiography or skeletal assessments were performed. The occlusion features of the primary dentition were evaluated using the method for the epidemiological registration of malocclusion by Bjoerk et al. [20].

Incisal overjet—the incisor relationship was assessed and measured by the horizontal distance between the incisal edge of the upper right central incisor and the buccal surface of the lower incisor. A range from 1 to 6 mm was regarded as normal and those more than 6 mm were regarded as increased. Reversed overjet was determined if the reversal was equal to or more than 2 mm; otherwise, it fell into the “edge-to-edge” category.

Canine relationship—the canine relationship was judged as a class I relationship if the tip of the maxillary canine occluded on the distal surface of the mandibular canine. The canine relationship would be classified as class II if the tip was mesial to the distal surface of the mandibular canine and class III if the tip was distal to that surface. Both left and right canine relationships were recorded separately.

Molar relationship—the relationship between the maxillary and mandibular primary second molars were recorded according to the vertical relationship between the distal surfaces of the two molars. The molar relationship would be classified as a mesial step if the distal surface of the maxillary molar lay mesial to that of the mandibular molars. A distal-step molar relationship refers to the situation where the distal surface of the maxillary molar is distal to that of the mandibular molars. A flush terminal plane was recorded when the distal surfaces of the maxillary and mandibular molars were on the same vertical line. Both left and right molar relationships were recorded separately.

Primate space (anthropoid space)—the maxillary primate space refers to the space between the maxillary lateral incisor and canine, and the mandibular primate space refers to the space between the canine and the first molar. The presence of the primary space was recorded when at least 2 mm of space were between the maxillary teeth and at least 1 mm was between the mandibular teeth. Both left and right maxillary and mandibular primate spaces were recorded separately.

Median diastema—median diastema was recorded if the space between the two maxillary primary central incisors was more than 1 mm. The measurement was made by inserting a 1 mm stainless steel wire between the two central incisors. 

Crossbite—anterior crossbite was recorded when one or more maxillary incisors and canines occluded to the lingual surfaces of the mandibular incisors. A posterior crossbite was recorded if one or more maxillary molars occluded buccal to the buccal surface of the mandibular molars. A unilateral left and right posterior crossbite and bilateral posterior crossbite were recorded.

Anterior open bite—anterior open bite was recorded if no vertical overlap was found between one or more maxillary and mandibular incisors when the molars were in the maximal intercuspal position.

Scissor bite—scissor bite was recorded when one or more maxillary molars were buccally located to the buccal surface of the mandibular molars. 

Five preschool children who were not participants of the survey were examined in a dental hospital prior to the survey as part of a calibration procedure that an experienced oral epidemiologist led. Approximately 10% of the children were re-examined during the survey to monitor the interexaminer reproducibility using the kappa statistic. 

Statistical analysis: The collected data were entered into a Microsoft Excel file for analysis using IBM SPSS Statistics 20 software (SPSS Inc., Chicago, IL, USA). Intra-examiner agreement in caries diagnosis was assessed by using Cohen’s Kappa statistics (SPSS Inc., Chicago, IL, USA). The distribution of the dental caries was not normal. The Mann-Whitney U test was used to analyze the differences between the caries experience (dmft) and groups with different groups of occlusion parameters. The caries prevalence between groups was assessed with a chi-squared test.

## 3. Results

A total of 538 kindergarten children from seven kindergarten classes were invited to participate in this study. Forty-one children had no parental consent form or were missing one, and two children were uncooperative to be examined and were thus excluded from the study. Therefore, 495 preschool children were examined. The response rate was 92% (495/538). Of these, 227 children (46%) were aged 4 years, and 264 children (53%) were boys. The value of the kappa statistic for the tooth status diagnosis was 0.95, and for the eight occlusal features mentioned above, it was in the range of 0.83 to 0.88. Approximately half (51%, *n* = 252) had no caries experience (dmft = 0). The overall mean (±SD) dmft score was 2.1 ± 3.6, with a mean dt = 2.0 ± 3.4. Gender and age were not significantly associated with either the mean dmft score or the studied occlusal features (*p* > 0.05).

Half (52%) of the children had a normal incisor relationship. One-third (38%) had increased overjet, and a minority (9%) had decreased overjet. Over three-quarters of the children had a class I canine relationship (79% for left canines, and 75% for right canines), followed by class III (16% for left canines, and 19% for right canines) and class II (5% for left canines, and 6% for right canines). More than half of the children had a flush terminal plane for their second molars (64% for left molars, and 65% for right molars), and a quarter had a mesial step (27% for left molars and 26% for right molars). The occlusion features of the children are shown in Table 1.

One-third (30%) had median diastema. Twenty percent (*n* = 101) had sizable mesial caries in at least the upper central incisor, making the assessment of diastema impossible. The other occlusion parameters could be assessed in most children. For children whose occlusion parameters could not be determined, this was due to untreated caries. Approximately a quarter of the children did not have primate spaces in the maxillary arch, and more than half of the children had reduced or no mandibular primate spaces. No statistically significant associations were detected between the occlusal features and the dental caries experience and prevalence. 

The features of malocclusion associated with the children are shown in Table 2. Approximately 12% of the children had anterior crossbite. Only approximately 1% of the children had unilateral posterior crossbite. No bilateral posterior crossbite or scissor bite was found. Mandibular displacement was recorded in approximately 5% of the children. No significant association between anterior crossbite and caries experience or caries prevalence was uncovered. In addition, no significant difference was identified in the other malocclusion features and caries experience. The number of cases was too small for statistical testing regarding malocclusion features and caries prevalence.

## 4. Discussion

The Government of Hong Kong provides fee assistance for kindergarten education, so almost all preschool children in Hong Kong go to kindergarten. However, attending kindergarten is not mandatory in Hong Kong, and some children could receive home schooling. The number of these children, however, is likely small and should not significantly affect the results of this study. Most preschool children attend a three-year kindergarten at 3 years of age in Hong Kong. Preschoolers in their first year (K1) of kindergarten begin to adapt to school life. The kindergarten environment is new, and they start to learn to leave their parents and mix with other children. As a result, they are often too young to cooperate for dental examinations in kindergarten. Children in K2 and K3 are 4 and 5 years old, respectively. They are familiar with school life, and thus, their cooperation is more likely. Moreover, all primary teeth should have erupted for caries and occlusal assessment.

This study dovetailed with an epidemiological survey [17] and used a random stratified cluster sampling technique. Clusters of children should theoretically be as heterogeneous as possible, and each cluster of children should be, to some extent, representative of the general population of children. In this study, the preschool children were chosen based on the kindergarten class they attended, and the use of this sampling method could reduce both time and cost. Nevertheless, the background of the children within a cluster might not be homogeneous, and the clusters might vary. Therefore, the variance of this cluster sample with a defined sample size could be larger than that of a simple random sample, and hence, the estimates are less precise. This study had a high response rate, which is common for studies involving the Chinese population [17,18]. This could be due to the emphasis on diligence and obedience in traditional Chinese culture, in addition to the social environment. Furthermore, a report on orthodontics, together with oral hygiene souvenirs, might have fostered the high response rate.

Non-parametric tests were used in this study because the data were not normally distributed. We did not make assumptions about parameters that could be dubious or untrue. However, non-parametric tests have less statistical power than parametric tests do. The interexaminer agreement on caries diagnosis was high. The clear-cut caries diagnosis at the cavitation level and the calibration exercise were the two main reasons for this. In addition, the interexaminer agreement on the assessment of the occlusal features was considered satisfactory, although it was not as high as that for the caries assessment. Apart from the bias of the examiners, the definitions of some occlusion parameters could be different among studies [21]. For example, the distance of each category of molar relationship (mesial step, flush terminal plane and distal step) could be different. It varied up to 2 mm for a flush terminal plane, depending on the examiners’ preferences in different studies [21]. In addition, the prevalence of a flush terminal plane decreases with age, and the prevalence of a mesial step increases with age, most likely due to the forward growth of the mandible [10]. The mesial-step molar relationship allows for a favorable intercuspation of erupting first permanent molars. Caution should be taken when comparing the data of this study with those of other studies.

The caries experience of the kindergarten children in Singapore (dmft = 1.5) is lower than that of the kindergarten children in this study [22]. Both Singapore and Hong Kong have implemented water fluoridation, and they are similar in terms of economic development. However, the caries experience was considerably lower when compared with Taiwan (dmft = 7.0) [23] and mainland China (dmft = 4.5) [24].

In this study, none of the occlusion parameters were associated with caries experience or prevalence. This finding supports the current view that dental caries has little association with the occlusion features of malocclusion [25]. This idea is contradictory to older studies reporting that malocclusion was associated with caries experience [26,27]. Dental caries was also reported to be associated with open bite and posterior crossbite [28,29]. It was hypothesized that a long duration of bottle feeding, mainly at night, could be a common risk factor for both dental caries, open bite, and posterior crossbite, excluding the possibility of a cause-effect relationship. However, this study did not find an association between anterior open bite and caries experience in Chinese preschool children. Caries experience was also suggested to be high in children with no spacing in the maxillary anterior teeth [30]. Still, Hong Kong is a city with water fluoridation, and fluoride toothpaste is readily accessible and widely used. It is not known if this could contribute to different findings.

The prevalence of the canine class II relationship of Chinese preschoolers is significantly lower (5% vs. 32%) than that of Danish children [31]. While ethnicity could be a reason for this, and the difference could be due to the inclusion of children with extracted teeth in the latter study. Although the prevalence of the class II canine relationship may decrease with advancing age due to the termination of some environmental factors, such as sucking habits in the older age group, this was not observed in this study. The mean overjet (1.9 mm) of the Chinese preschoolers is similar to that of the study conducted in India [10]. 

Open bite is often associated with sucking habits, such as the long-term use of a pacifier, bottle feeding, and finger sucking. This malocclusion was found in only 1% of the children, and this prevalence is different from that reported in other studies [10,31]. A study may be warranted to examine the effect of sucking and other unfavorable oral habits on open bite. The prevalence of anterior crossbite in the study is higher than that in Saudi and English children. The prevalence of posterior crossbite in the present sample is 1%, which is lower than that in another study [32]. The Caucasian population generally has a higher prevalence of posterior crossbite than the African and Asian populations [32].

Although this study found no association in the preschool children of caries experience with occlusal and malocclusion features, severe caries in primary teeth may affect the development of the permanent dentition. A study reported a positive association between midline deviation and the permanent dentition, as well as between severe caries and the primary dentition [33]. In this study, caries on the primary incisors made it difficult to determine the presence of median diastema. Hence, the results of this study should be interpreted with caution. It is essential for a dentist to detect any anomalies that can predispose the malocclusion development of the permanent dentition.

## 5. Conclusions

Most Hong Kong Chinese preschool children had a normal occlusion. Median diastema was found in 30% of the children. Twelve percent of the children had anterior crossbite, and other malocclusion features were not common. Approximately half had caries experience, which was not associated with their occlusal or malocclusion features.

## Figures and Tables

**Table 1:** Occlusal features and caries of Hong Kong preschool children.

Occlusal Feature	Frequency (%)	Caries Experience		Caries Prevalence	
		dmft ± SD	*p* Value	n (%)	*p* Value
Incisal overjet (*n* = 481)					
Normal	212 (52%)	1.9 ± 3.3	0.514	105 (51%)	0.481
Increased	184 (38%)	2.0 ± 3.3		84 (41%)	
Decreased	45 (10%)	1.4 ± 2.6		18 (8%)	
Left canine relationship (*n* = 491)					
Class I	386 (79%)	2.0 ± 3.4	0.763	169 (78%)	0.981
Class II	26 (5%)	1.8 ± 3.0		11 (5%)	
Class III	79 (16%)	2.3 ± 4.0		36 (17%)	
Right canine relationship (*n* = 485)					
Class I	367 (75%)	1.9 ± 3.4	0.184	160 (76%)	0.459
Class II	28 (6%)	3.1 ± 4.0		15 (7%)	
Class III	90 (19%)	1.8 ± 3.2		37 (17%)	
Left molar relationship (*n* = 487)					
Flush terminal plane	314 (64%)	1.9 ± 3.3	0.806	132 (63%)	0.737
Distal step	44 (9%)	1.9 ± 2.8		20 (9%)	
Mesial step	129 (27%)	2.1 ± 3.7		59 (28%)	
Right molar relationship (*n* = 487)					
Flush terminal plane	315 (65%)	1.8 ± 3.1	0.416	132 (63%)	0.342
Distal step	45 (9%)	2.5 ± 3.4		24 (11%)	
Mesial step	127 (26%)	2.1 ± 3.7		55 (26%)	
Left upper primate space (*n* = 479)					
Presence	375 (79%)	1.7 ± 2.8	0.171	184 (77%)	0.666
Absence	104 (21%)	2.3 ± 4.0		53 (23%)	
Right upper primate space (*n* = 495)					
Presence	366 (76%)	1.8 ± 3.0	0.935	182 (77%)	0.819
Absence	113 (24%)	1.8 ± 3.1		54 (23%)	
Left lower primate space (*n* = 487)					
Presence	219 (45)	1.9 ± 3.3	0.732	106(43%)	0.338
Absence	268 (55)	2.0 ± 3.3		142 (57%)	
Right lower primate space (*n* = 484)					
Presence	226 (47)	2.0 ± 3.4	0.598	109 (45%)	0.592
Absence	258 (53)	1.9 ± 3.1		136 (55%)	
Median diastema (*n* = 404)					
Presence	130 (30)	1.3 ± 2.6	0.126	48 (34%)	0.688
Absence	274 (70)	1.0 ± 2.1		93 (66%)	

**Table 2:** Malocclusion features and caries of Hong Kong preschool children.

Malocclusion Feature	Frequency (%)	Mean dmft ± SD	*p* Value
Anterior crossbite (*n* = 493)			
Presence	58 (12)	1.7 ± 3.1	0.352
Absence	435 (88)	2.2 ± 3.6	
Anterior open bite (*n* = 493)			
Presence	4 (1)	1.0 ± 1.2	0.920
Absence	489 (99)	2.1 ± 3.6	
Unilateral left posterior crossbite (*n* = 493)			
Presence	6 (1)	3.0 ± 4.3	0.607
Absence	487 (99)	2.1 ± 3.6	
Unilateral right posterior crossbite (*n* = 493)			
Presence	5 (1)	3.0 ± 4.6	0.492
Absence	488 (99)	2.1 ± 3.6	
Bilateral posterior crossbite (*n* = 493)			
Presence	0 (0)	-	-
Absence	493 (100)	2.1 ± 3.6	
Scissor bite (*n* = 493)			
Presence	0 (0)	-	-
Absence	493 (100)	2.1 ± 3.6	
